# Social Justice and Social Order: Binding Moralities across the Political Spectrum

**DOI:** 10.1371/journal.pone.0152479

**Published:** 2016-03-31

**Authors:** Ronnie Janoff-Bulman, Nate C. Carnes

**Affiliations:** Department of Psychological and Brain Sciences, University of Massachusetts, Amherst, Massachusetts, United States of America; University of Vermont, UNITED STATES

## Abstract

Two studies explored the relationship between political ideology and endorsement of a range of moral principles. Political liberals and conservatives did not differ on intrapersonal or interpersonal moralities, which require self-regulation. However differences emerged on collective moralities, which involve social regulation. Contrary to Moral Foundations Theory, both liberals and conservatives endorsed a group-focused binding morality, specifically Social Justice and Social Order respectively. Libertarians were the group without a binding morality. Although Social Justice and Social Order appear conflictual, analyses based on earlier cross-cultural work on societal tightness-looseness suggest that countries actually benefit in terms of economic success and societal well-being when these group-based moralities co-exist and serve as counterweights in social regulation.

## Introduction

Political ideologies reflect distinct underlying moral principles. In the studies below we explore morality through the lens of political ideology in an effort to better understand where the political left and right actually differ, and the implications of these differences. More specifically, where do the moralities of liberals and conservatives diverge, and are their moral principles necessarily antagonistic? Might they instead reflect distinct yet complementary orientations to social problem-solving that, removed from politics, serve societies well? To begin to address these questions, we first turn to a map of the moral domain.

### Model of Moral Motives (MMM)

There are two broad but distinct ways of being moral: engaging in positive, selfless behaviors or restraining negative, self-interested behaviors. These are motivationally distinct, for the former involves the need to establish new “good” motivations, whereas the latter involves inhibiting pre-existing “bad” motivations. In drawing this distinction, Janoff-Bulman, Sheikh and Hepp [1] labelled these two broad orientations prescriptive morality (*the shoulds*) and proscriptive morality (*the should nots*), and noted that they reflected differences between approach and avoidance applied to the moral domain. Keeping self-interest in check (e.g., restraining the temptation to lie or cheat) is clearly moral, but it is not the same as actually helping another—that is, not harming is not equivalent to helping. Proscriptive and prescriptive morality represent two distinct motivations: proscriptive morality relies on behavioral inhibition to restrict the “bad,” whereas prescriptive morality involves behavioral activation to enable the “good.”

**Fig 1:**
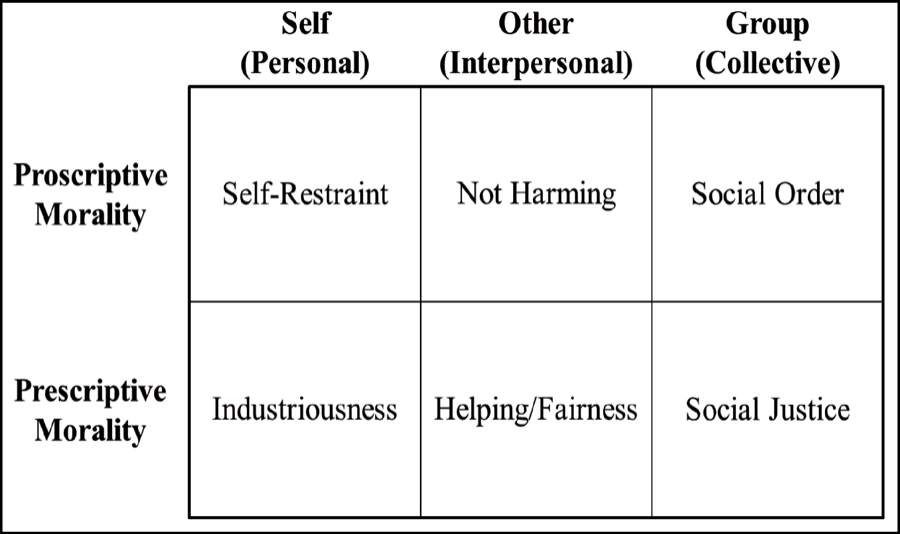
Model of Moral Motives.

In our recent effort to map the moral domain, we relied on this motivational distinction to generate discrete moral motives [2,3]. More specifically, we crossed proscriptive and prescriptive morality with the three primary domains of behavior studied by psychologists—the intrapersonal (self), the interpersonal (another), and the collective (group). As evident in Fig 1, the resulting six cells are: Self-Restraint, Industriousness, Not Harming, Helping/Fairness, Social Order, and Social Justice. In the first column are the self-focused moral motives. Here MMM identifies Self-Restraint (proscriptive) and Industriousness (prescriptive) as intrapersonal moral motives, involving moderation and attributes often associated with the “protestant ethic” respectively. Although these motives focus on the self, they involve distal benefits for group survival in countering wastefulness (i.e., via moderation) and contributing to the groups’ resources and competencies (i.e., via hard work, persistence). Recent research by Hofmann and colleagues [4] on moral acts in everyday life provides support for the claim that such self-directed moral principles should be included in any comprehensive model of morality.

The center (interpersonal) column of MMM [2] refers to our interactions with specific, identifiable others, and the two cells—Not Harming and Helping/Fairness—reflect the prototypes of morality for both proscriptive and prescriptive regulation [5]. Not harming involves not only refraining from physically hurting another, but inhibiting behaviors such as lying and stealing that would otherwise advantage the actor. Helping entails providing for the well-being of another; fairness, too, is a prescriptive moral motive in the interpersonal domain and involves giving others their due. The close relationship between helping and fairness are evident in their early integration in the phenomenon of reciprocal altruism.

All of morality ultimately involves the facilitation of group living and group survival, but the proximal behaviors need not focus specifically on the group, as evident from the preceding four cells of MMM. However, the third and final column of MMM focuses specifically on the collective and entails broad social regulation. As Tomasello and Vaish [6] write, the evolution of morality involves a two-step process: “…the first step is mutualistic collaboration and prosocially motivated interactions with specific individuals, and the second step is the more abstract, agent-neutral, norm-based morality of individuals who live in more large-scale cultural worlds full of impersonal and mutually known conventions, norms, and institutions” (p. 232).

There are two group moral motives in MMM: Social Order (proscriptive) and Social Justice (prescriptive). Most generally, Social Order is in the service of collective coordination, and is particularly responsive to dangers and threats to the group, whether physical threats to safety or psychological threats to identity. A Social Order morality emphasizes the importance of group conformity and strict adherence to behavioral norms; self-interest and individual self-expression are constrained in the service of the larger group’s interests. Social Justice involves communal responsibility and activates collective efforts to advance the group’s welfare. There is a particular focus on equality-based distributional justice. Most generally, a Social Order morality is oriented towards protecting the group, whereas a Social Justice morality is oriented towards providing for the group [2,7].

### Two Group Moralities

In this paper we focus on the two group moralities of MMM, for we believe they address a provocative issue raised by Moral Foundations Theory (MFT). More specifically, Haidt and colleagues [8–11] in the dominant model of morality to date (MFT), posit five moral foundations. There are two “individualizing” foundations—Harm/Care and Fairness/Reciprocity—where the individual is the focus of moral concern; these involve “individual-focused contractual approaches to society” (p. 369) [8]. In addition there are three “binding” foundations—Ingroup/Loyalty, Authority/Respect, and Purity/Sanctity—that bind people together; their focus of moral concern is the group [8].

Using MFT, Haidt and colleagues [8,12–14]) note that liberals emphasize the individualizing foundations, whereas conservatives rely on both the individualizing and binding foundations—that is, conservative morality is based in all five foundations, whereas liberal morality is based in only two. The provocative implication of these assertions is that liberals don’t have a morality that is focused on the group; this is the provenance of only conservatives.

Yet the binding (i.e., group) moralities in MFT are all aspects of a Social Order morality; they are mechanisms in the service of a binding proscriptive moral regulation [2,3]. Haidt and colleagues overlook a prescriptive group morality—Social Justice. We contend their conclusion that only conservatives have a group morality is a consequence of failing to include a group morality that might represent liberal (versus conservative) moral motives.

It is important to address possible reactions claiming that Social Justice is really the same as Fairness/Reciprocity, which is included in MFT. Although they may be used interchangeably in everyday conversation, they are nevertheless distinct constructs [2,3 for a more detailed discussion]. Paralleling the important distinction between microjustice and macrojustice by Brickman, Folger, Goode, and Schul [15], fairness is based in considerations of another’s deservingness and involves identifiable others whose inputs or attributes are assessed. In contrast, social justice is based in considerations of the distribution of resources across a group; it is deindividuating and focused on the group or, more specifically, others as categorical members of the group [16,17 for similar distinctions]. As in MMM, Fairness is appropriately regarded as an individualizing foundation in MFT, and considerable past research has found minimal liberal-conservative differences in this moral foundation. In contrast, we believe Social Justice is endorsed far more by liberals than conservatives.

It might also be argued that Social Justice is really the same as care, as represented by the Harm/Care foundation in MFT [18]. To some extent, from a sufficiently distal perspective, all of morality is about harm and care [5,19], yet individuated Care entails known, identifiable others and assessment of need, whereas Social Justice is about the form of a distribution across a group and its fit with equality concerns. Again, Social Justice is a binding, group-based morality, whereas Harm/Care is an individualizing morality endorsed by both liberals and conservatives.

Contrary to MFT, our claim is that there *is* a group-based morality for liberals, and that it is Social Justice. More specifically, we propose two distinct group moralities—Social Order and Social Justice, with the former positively associated with political liberalism and the latter with political conservatism. Liberals, in other words, do have a group morality; it simply hasn’t been included in MFT, the dominant model of morality to date.

### Self-Regulation versus Social-Regulation

What might account for the hypothesized positive associations between conservatism and Social Order and between liberalism and Social Justice? To answer this question, we can turn to differences in fundamental motivational orientation. Here we draw from past work to suggest that an avoidance motivation is dominant for conservatives, whereas an approach motivation is dominant for liberals. More specifically, past research has found that conservatives are more reactive to threat, display greater disgust sensitivity, have greater category restrictiveness, are more apt to be the product of restrictive parenting, focus on losses rather than gains and show a general negativity bias; in contrast, liberals exhibit greater openness to experience, engage in more exploratory behaviors, are more apt to be the product of egalitarian parenting, and focus on gains rather than losses [20–34]. Conservatives’ greater avoidance orientation and liberals’ greater approach orientation provide a basis for the proposed differences found in group morality, because Social Order is a proscriptive avoidance-based moral motive, whereas Social Justice is a prescriptive, approach-based moral motive.

The distinct motivational proclivities of liberals and conservatives may account for differences in the group moralities, but raise a different but related question. Given the expected differences in the binding group morality of liberals versus conservatives (i.e., Social Justice and Social Order), it seems important to consider why the moral differences are expected to arise in this domain and not the others (i.e., self or interpersonal). This expectation is consistent with the MFT finding of few differences in the individualizing foundations based on political ideology. How might we account for the expected differences in Social Justice and Social Order, but not the other four cells of MMM?

The self and interpersonal cells are guides for how we should act (or refrain from acting) with regard to ourselves and specific, identifiable others. They are about self-regulation—regulating individual behavior with regard to the self or another. The group moral motives are guides for how we should act with regard to the group, but more important, they are broadly about how collectives should be run and monitored. This is the domain of social regulation. In the case of self-regulation, the appropriate level of analysis is clearly the individual, and there is typically some “balance” within the individual. That is, regardless of political orientation, we rely on both proscriptive and prescriptive morality. A moral person would be high on both Self-Restraint and Industriousness, and on both Helping/Fairness and Not Harming. Even a motivational proclivity for one or the other would not preclude considerable reliance on the other. In fact a strong over-reliance on one or the other form of moral regulation is likely to be maladaptive when we are dealing with self-regulation [35].

When it comes to self-regulation, we can rely on both systems for maximal flexibility, and greater morality in one system is likely to be associated with being more moral in the other. Yet when we are considering social regulation, the level of analysis is now the group or collective. Balance may still be desirable for societal adaptability, but now it is can be achieved across individuals as opposed to within individuals. More specifically, an effective way to maintain balance would be for some subpopulations to espouse one group-based morality and others to advocate the other group-based morality. In this way society can rely on both systems (i.e., proscriptive and prescriptive—Social Order and Social Justice) for maximal adaptability.

From this perspective Social Order and Social Justice essentially serve as counterweights, maximizing the possibility of positive group outcomes by affording maximal flexibility in dealing with collective concerns. Is balance in these group-based moralities associated with societal success? We explore this possibility in Study 3; cross-cultural data on tight versus loose societies [36] provide a basis for our analyses. First, in Studies 1 and 2 we explore the relationship between moral motives and political orientation, with a particular focus on the group-based moralities. In doing so we also present the MMM scale, which assesses the moral motives in the six cells of the model.

## Study 1

To determine the role of the proposed moral motives in regulating human behavior and group living, we developed a scale to assess the moral motive of MMM. Scale development began with ten items per cell. Following multiple iterations of distribution and analysis, we arrived at a final version of the MMM scale. Here we first present the results of a confirmatory factor analysis to provide support for subsequent use of the scale. Then, we use structural equation modeling to assess the relationship between political orientation and the six moral motives. Given the common reliance on the individualizing foundations by both liberals and conservatives, we did not expect differences on the interpersonal cells of the model, nor did we expect differences on the intrapersonal motives. We did, however, expect political orientation to be associated with two distinct group-based moralities, with conservatism associated with Social Order and liberalism with Social Justice.

### Method

#### Participants

Study participants were 311 individuals recruited through Amazon Mechanical Turk. Of these 200 (64%) were male and 111 (36%) were female, and 46 (15%) identified as Asian, 21 (7%) as Black, 15 (5%) as Hispanic, 222 (71%) as White, and 6 (2%) as Other. The mean age was 32.87, with a range of 18 to 74; the median age was 28.

#### Measures

The 30-item Model of Moral Motives (MMM) Scale assesses the six moral motives with five items each (see Appendix for all items). All items were rated on a 7-point scale anchored from “strongly disagree” (1) to “strongly agree” (7). Three parcels were constructed to represent each moral motive using the item-to-construct balance approach (Little et al., 2002). Sample items include: “Life is full of unhealthy attractions, so it’s necessary for me to develop a strong sense of self-discipline and control” (Self-Restraint); “When things get tough, I apply myself and work even harder to overcome difficulties” (Industriousness); “It is always wrong to kill another human being” (Not Harming); “When someone does me a favor, I try particularly hard to return the favor” (Helping/Fairness); “In a good society, there must be very little deviation from behaviors viewed as appropriate” (Social Order); and “It is important for those who are better off to help provide resources for the most vulnerable members of society” (Social Justice).

Political orientation was assessed in the demographics questionnaire with one 7-point Likert-type rating scale anchored from “very liberal” (1) to “very conservative” (7). Participants completed the MMM scale online and then completed a demographic questionnaire. Participants were then debriefed and provided a code for payment. (See S1 File for all Study 1 data.)

### Results

We first created a CFA model in LISREL 8.80 to test how well six latent factors (i.e., Self-Restraint, Industriousness, Not Harming, Helping/Fairness, Social Order, and Social Justice) represent the 18 parceled indicators. We loaded the three indicators corresponding to each construct on their respective factors and standardized the factor variances. The model also included 18 indicator error variances and 15 factor covariances. Although the model chi-square was significant (*χ*^*2*^_*M*_(120) = 273.68, *p* < .001), the values for the other recommended fit indices were excellent (*RMSEA* = .065, *CFI* = .973, *SRMR* = .047). In addition, an inspection of the standardized parameter estimates revealed that every single indicator loaded strongly on its corresponding latent factor (see Table 1). The correlations between the six latent factors are depicted in Table 2. It is interesting to note that while both the self-focused moral motives and the other-focused moral motives were positively correlated (*ϕ*_self_ = .544, t = 11.74, p < .05; *ϕ*_other_ = .746, t = 17.62, p < .05), the group-focused moral motives of Social Order and Social Justice were negatively correlated with each other (*ϕ*_group_ = -.181, t = 3.06, p < .05).

**Table 1:** Estimated Standardized Factor Loadings in MMM.

Indicator	*λ*	*t-value*	*p-value*
Self-Restraint1	.83	17.46	< .05
Self-Restraint2	.88	19.14	< .05
Self-Restraint3	.90	19.77	< .05
Industrious1	.82	16.89	< .05
Industrious2	.86	18027	< .05
Industrious3	.88	18.91	< .05
Not Harming1	.76	14.12	< .05
Not Harming2	.65	11.73	< .05
Not Harming3	.77	14.32	< .05
Helping/Fairness1	.81	16.03	< .05
Helping/Fairness2	.74	14.37	< .05
Helping/Fairness3	.72	13.66	< .05
Social Order1	.85	18.32	< .05
Social Order2	.86	18.62	< .05
Social Order3	.94	21.21	< .05
Social Justice1	.93	21.07	< .05
Social Justice2	.89	19.94	< .05
Social Justice3	.91	20.43	< .05

All parameter estimates are single-factor loadings on corresponding latent factors.

**Table 2:** Estimated Correlations between Latent Factors in MMM.

	Restraint	Industrious	Not Harming	Helping	Social Order	SocialJustice
Restraint						
Industrious	.544*					
Harming	.375*	.464*				
Helping	.495*	.604*	.746*			
Order	.305*	.102	.102	.016		
Justice	.146*	.229*	.390*	.555*	-.181*	

* p < .05.

Next, considering the claim made by Graham [18] that Social Justice is subsumed under Care and Fairness, we explicitly tested how well a five-factor model (with just one factor to represent the Social Justice and Helping/Fairness indicators) would fit the data compared with a six-factor model. We collapsed Helping and Social Justice into a single latent factor by constraining the correlation between them to one and constraining all the other correlations with Social Justice to zero, which is equivalent to creating a model with all the indicators loading on a single Helping latent factor [37,38]. The resultant five-factor model was a poor fit to the data (*χ*^*2*^_*M*_(125) = 529.01, *p* < .001, *RMSEA* = .101, *CFI* = .930, *SRMR* = .161), and was a significantly worse fit to the data compared with the six-factor model (∆*χ*^*2*^(5) = 255.33, *p* < .001). This suggests that Social Justice and Helping/Fairness are distinct constructs.

Finally, having established our measurement model, we created a structural equation model testing how political orientation is related to the six moral motives in the measurement model. We loaded the single political orientation indicator on its own latent construct of Politics and its error variance was fixed to zero. We created a model in which the Politics factor predicted the six moral motive factors. Although the model chi-square was significant (*χ*^*2*^_*M*_(132) = 293.34, *p* < .001), the values for the other recommended fit indices were again excellent (*RMSEA* = .063, *CFI* = .974, *SRMR* = .047). An inspection of the structural model revealed that Politics (with higher scores indicating greater conservatism) was positively correlated with Social Order (γ = .547, t = 6.28, p < .05) and negatively correlated with Social Justice (γ = -.647, t = 6.86, p < .05) as predicted. In contrast, Politics was not significantly correlated with Industriousness (γ = .075, t < 1), Self-Restraint (γ = .123, t = 1.58), Helping/Fairness (γ = -.091, t < 1), or Not Harming (γ = -.061, t = 1.10).

### Discussion

Study 1 validated the MMM scale using confirmatory factor analysis; the 30 items in the MMM scale appear to represent six latent constructs corresponding to the moral motives hypothesized in MMM. In addition, as predicted, political orientation was associated with the group-based moral motives but not the self-focused or other-focused moral (i.e., individualizing) motives. As expected, Social Order was positively associated with political conservatism, whereas Social Justice was negatively associated with conservatism (i.e., positively associated with political liberalism). Interestingly, the self-focused and other-focused moral motives were positively correlated, whereas the group-based moral motives were negatively correlated. More specifically, in the domain of self-focused and other-focused motives, the domains of self-regulation, endorsement of prescriptive and proscriptive morality is positively associated; the higher a person is on one, the higher s/he is likely to be on the other. In the domain of social regulation, the pattern reverses. Proscriptive and prescriptive moral motives (i.e., Social Order and Social Justice) are negatively correlated—the higher on one, the lower on the other, suggesting that at the level of social regulation, the two moral motives are represented by different societal groups.

## Study 2

Study 2 was conducted in order to replicate and extend the findings of Study 1. Specifically, we were interested in the relationship between political orientation and the moral motives. Just as in Study 1, we hypothesized that political orientation would primarily be associated with the group-based moral motives, with Social Order associated with conservatism and Social Justice associated with liberalism.

### Method

#### Participants

Study participants were 295 individuals recruited through Amazon Mechanical Turk. There were 184 (62%) males and 111 (38%) females. Of these 223 (74%) identified as White, 23 (8%) as Asian, 19 (6%) as Black or African-American, 24 (8%) as Hispanic, and 6 (2%) as Other. The mean age of participants was 38.33, with a range of 18 to 67; the median age was 28.

#### Measures

All participants completed the 30-item MMM scale (see Appendix). All six five-item composite variables were adequately reliable: Self-Restraint (*α* = .913), Industriousness (*α* = .844), Not Harming (*α* = .722), Helping/Fairness (*α* = .777), Social Order (*α* = .928), and Social Justice (*α* = .918).

Political orientation was assessed in the demographics with two 7-point Likert-type rating scales anchored from “very liberal/strong democrat” (1) to “very conservative/strong republican” (7). The liberal/conservative and Democrat/Republican scales were highly correlated (r(293) = .844) and were combined into a single political orientation scale, with higher scores indicating greater conservatism. In addition, participants indicated whether they identified as Democrat (yes/no), Republican (yes/no), Libertarian (yes/no), or none of the above (yes/no). (See S2 File for all Study 2 data.)

### Results

The mean on the 7-point political orientation scale was 3.15 (*SD* = 1.49), indicating a somewhat liberal sample. However, only 49% of the sample (*n* = 143) identified as Democrats; 16% (*n* = 47) identified as Republicans, 14% (*n* = 42) as Libertarians, and 26% (*n* = 78) as none of the above. Participants could identify with more than one group, and 9 of the Libertarians also identified as Democrats and 5 as Republicans.

We conducted a multiple regression with the six moral motives included as predictors of political orientation. As predicted, Social Justice was negatively associated with political orientation (*B* = -.365, *SE* = .063, *t*(294) = 5.80, *p* < .001, *η*_*p*_^*2*^ = .105) whereas Social Order was positively associated with political orientation (*B* = .353, *SE* = .052, *t*(294) = 6.73, *p* < .001, *η*_*p*_^*2*^ = .136). Once again none of the intrapersonal or interpersonal moral motives were significantly associated with political orientation: Self-Restraint (*t* = 1.22, *p* = .225), Industriousness (*t* < 1), Not Harming (*t* < 1), or Helping/Fairness (*t* < 1). These findings replicate the Study 1 findings.

Furthermore, political identification as Democrat, Republican, or Libertarian supported these differences on the two group-based moralities. These political groups differed on liberalism/conservatism (*F*(2, 214) = 235.97, p < .001, *η*_*p*_^*2*^ = .688), and post hoc analyses indicated that each differed significantly from other two (all *p* < .001); the respective means for Democrats, Libertarians, and Republicans were 2.04 (SD = .81), 3.48 (SD = 1.20), and 5.54 (SD = .95). There were group differences on Social Justice (*F*(2,214) = 16.13, *p* < .001, *η*_*p*_^*2*^ = .131) and Social Order (*F*(2,214) = 19.53, *p* < .001, *η*_*p*_^*2*^ = .154). Post hoc analyses indicated that Democrats were significantly higher than Libertarians and Republicans on Social Justice (all *p* < .001, *M*_*DEM*_ = 5.56, SD = 1.03; *M*_*LIB*_ = 4.80, SD = 1.34; *M*_*REP*_ = 4.45, SD = 1.58), whereas Republicans were significantly higher than Democrats and Libertarians on Social Order (all *p* < .001, *M*_*REP*_ = 4.48, SD = 1.44; *M*_*DEM*_ = 3.01, SD = 1.42; *M*_*LIB*_ = 2.84, SD = 1.36). There were no significant differences for any of the other moral motives. Interestingly, Democrats and Republicans were higher on one or the other of the binding group moralities; it was Libertarians who were lower on both.

The results for analyses involving the three groups—Democrats, Republicans, and Libertarians—were the same regardless of whether the Libertarians who also identified as Republicans or Democrats were coded as one of the latter two groups (leaving those who identified only as Libertarians as members of this group) or were coded solely as Libertarians. Results reported here code dual identification as Libertarians. When coded instead as Democrats or Republicans, the three groups again remain significantly different from one another, with respective liberal/conservative scores changing very little (2.05 for Democrats, 3.68 for Libertarians, and 5.51 for Republicans), no doubt accounting for the equivalent findings regardless of coding. In addition, the same differences arose across the groups when a fourth group was added to analyses—those who did not identify with any of the three political groups (n = 82). These politically unidentified participants looked like liberals on Social Justice (M = 5.00, which was not significantly different from Democrats, but was significantly different from Republicans and Libertarians). On Social Order (M = 3.38) they looked like Democrats and Libertarians in that they did not significantly differ from these groups, but significantly differed from Republicans.

### Discussion

Differences in liberalism and conservatism were not associated with differences in the intrapersonal or interpersonal moral motives of MMM. Echoing findings from work by Haidt, Graham, and colleagues [7–9,12,14] on MFT, it was in the group domain—the realm of binding morality—that differences emerged. However, rather than demonstrate that conservatives have a group-based morality and liberals do not, the current studies suggest instead that there is a binding group morality associated with each political orientation—Social Order for conservatives and Social Justice for liberals.

As would be expected, those who identified as Democrats or Republicans differed considerably on political orientation, with Democrats significantly more liberal and Republicans significantly more conservative. The study also included the option of identifying as Libertarian, and analyses found that these individuals looked like liberals when considering Social Order and like conservatives when considering Social Justice. Interestingly, they don’t seem to endorse either group morality strongly, suggesting that it is libertarians, and not liberals, who lack a group-based morality. This finding is consistent with the view that libertarians are highly individualistic. The absence of a strong group-based morality is evident in libertarians’ endorsement of personal autonomy in both the economic and social domains, resulting in both fiscal conservatism and social liberalism [6].

## Study 3

Given that conservatism is associated with Social Order, and liberalism with Social Justice, is there some benefit to the presence of both orientations in a society? Is their co-existence adaptive in some way? The adaptive value of balance in self-regulation might suggest similar benefits in the realm of social regulation. Given that morality functions to facilitate social living and group survival [2,39–42], the two binding moralities may help a society address different problems (e.g., threats, resource distribution) in distinct ways (e.g., coordination versus cooperation), thereby providing maximal flexibility in addressing collective challenges and concerns [43]. Is a balance of Social Order and Social Justice associated with greater success for a given society? Do Social Order and Social Justice, in other words, serve as adaptive counterweights?

In order to provide a preliminary test of this hypothesis, we turned to the excellent cross-cultural work of Gelfand and colleagues [36,44] regarding tight versus loose societies. They define tight societies as those that have very strong norms, low tolerance for deviant behavior, and strict punishment for norm violations. Loose societies, in contrast, have weaker social norms and a high tolerance for deviant behavior, as well as greater civil liberties and civil rights. It is probably apparent to the reader that tight societies are those that value Social Order, and Gelfand did find that Tightness is positively associated with conservatism [45]. Interestingly, Gelfand et al. [36] found that these societies have experienced greater ecological and historical threats than loose societies. This would be consistent with a proscriptive orientation focused on protection from harm and the need for coordination in the face of societal challenges—and thus a Social Order group morality. Loose societies were far less likely to have experienced such threats, and their greater openness and tolerance seem associated with a group morality more akin to Social Justice. Importantly, Gelfand et al. [45] found a negative correlation (-.41) between tightness and egalitarian commitment in their international sample. Although Social Order and Social Justice may have varying relationships across countries, the overall pattern of findings suggests that Social Order and Social Justice, represented by tightness and egalitarian commitment, are nevertheless likely to be supported by different segments of the population within a given society.

Gelfand and colleagues assessed tightness and looseness in 33 countries. Economic success was not a focus of their research, but they nevertheless retrieved economic indicators (i.e., gross domestic product per capita and global competitive scores) and found these were unrelated to tightness-looseness. In testing these relationships, Gelfand et al. looked at the linear relationships and found no associations. However, if balance is best we would expect a significant curvilinear relationship, with countries in the middle—representing a balance of looseness and tightness—evidencing greatest success. We do not mean to suggest that economic production is the best measure of success, however, so we also retrieved general happiness scores for these countries. Here too we expected to find a significant curvilinear relationship reflecting the benefits of balance in group-based moralities.

### Method

We used the tightness-looseness scores reported by Gelfand et al. [36] for the 33 countries they studied. (Because there are no current economic or happiness indicators that differentiate between the former East and West Germany, we computed a weighted tightness score for all of Germany that accounts for differences in sample size. Thus, our analyses are based on 32 countries.) The Gelfand et al.data were collected between 2000 and 2003, so we retrieved GDP per capita and Global Growth Competitiveness Index rankings for 2003 from the World Bank [46] and World Economic Forum [47] respectively. We retrieved a measure of subjective well-being from the World Happiness Report [48], a landmark study launched by the United Nations to assess subjective well-being across nations. The first World Happiness Report was published in 2012, and the 2014 results reported in the 2015 report actually represent combined findings from 2012, 2013, and 2014. The happiness measure is a composite of high positive affect, low negative affect, life satisfaction, and meaning in life. To ensure that the use of this more recent data did not produce anomalous findings with respect to tightness-looseness, we also retrieved GDP per capita for 2014. (See S3 File for all Study 3 data.)

### Results and Discussion

**Fig 2:**
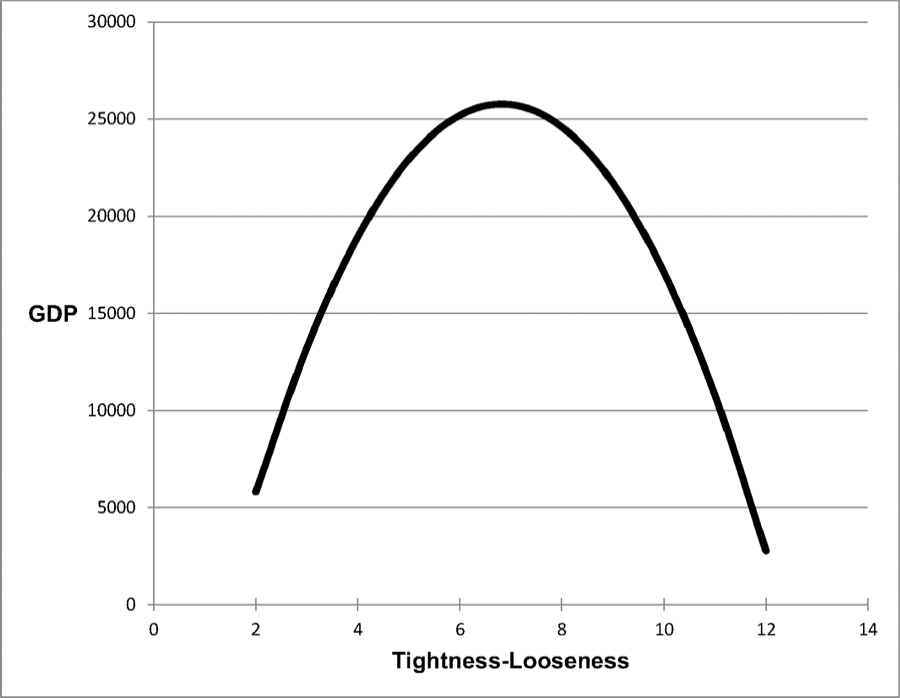
Tightness-Looseness and GDP per capita (2003).

**Fig 3:**
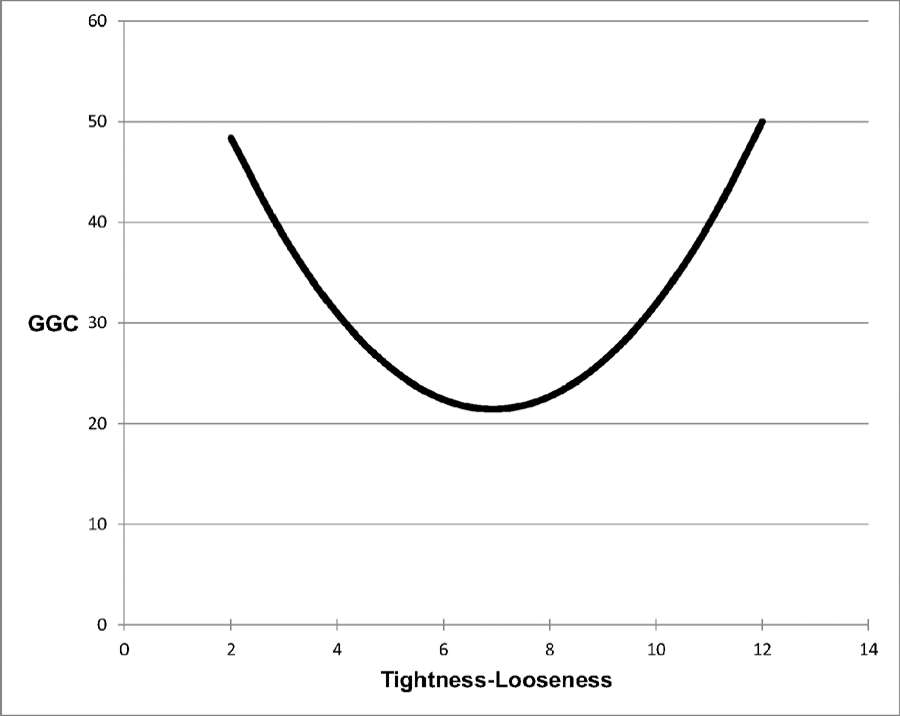
Tightness-Looseness and Global Growth Competitiveness Index (2003).

We first ran linear regressions and, replicating the findings of Gelfand and her colleagues, found no association between tightness-looseness and 2003 GDP per capita (*B* = -179.46, *SE* = 899.24, *t*(30) = -.20, *p* = .843) or between tightness-looseness and 2003 Global Growth Competitiveness rankings (*B* = .06, *SE* = 1.39, *t*(30) = .04, *p* = .965). We then ran these regressions with the quadratic terms to determine whether there is a curvilinear relationship between tightness-looseness and these economic indices. The quadratic parameter predicting 2003 GDP per capita was significant (*B* = -858.38, *SE* = 258.64, *t*(30) = -3.32, *p* = .002, *η*_*p*_^*2*^ = .275). The inverted U-shaped curve, with greatest GDP per capita represented by those in the middle of the tightness-looseness continuum, is apparent in Fig 2. The quadratic parameter for the 2003 Global Growth Competitiveness index was significant as well (*B* = 1.11, *SE* = .42, *t*(30) = 2.65, *p* = .013, *η*_*p*_^*2*^ = .195), with the most balanced countries being the most competitive (see Fig 3); the most competitive country is ranked 1, and thus the curve is U-shaped and not inverted.

**Fig 4:**
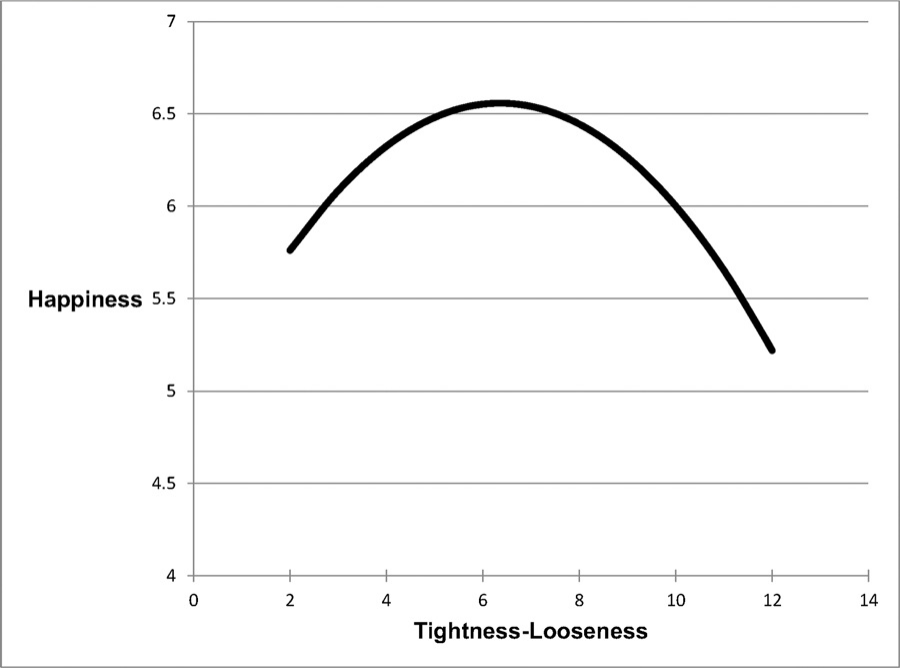
Tightness-Looseness and Happiness (2014).

We next ran a linear regression to test whether there is a curvilinear relationship between tightness-looseness and 2014 happiness. Once again, the quadratic parameter predicting happiness was significant (*B* = -.042, *SE* = .019, *t*(30) = -2.24, *p* = .033, *η*_*p*_^*2*^ = .148). As can be seen in Fig 4, the inverted U-shaped curve suggests those in the middle of the tightness-looseness continuum are the happiest. To ensure that 2014 was not unusual relative to our 2003 findings, we conducted a final linear regression testing whether there is a curvilinear relationship between tightness-looseness and 2014 GDP per capita. The analysis revealed the exact same pattern of relationships as in 2003 with a significant quadratic parameter (*B* = -1090.26, *SE* = 430.92, *t*(30) = -2.53, *p* = .017, *η*_*p*_^*2*^ = .181), suggesting once again that a balance between tightness and looseness is healthiest for a society.

Successful societies are those with a balance of tightness and looseness—those in the middle of the continuum. The translation from tightness and looseness to Social Order and Social Justice is certainly not perfect, and thus this is a somewhat crude first test. It is certainly possible that balance can be achieved by having most people in the center, advocating some moderate level of both group moralities. However, this would suggest little socio-political disagreement in societies, which does not appear to be descriptive of political realities across the globe. In addition, in their multi-nation sample, Gelfand and colleagues found that tightness and egalitarianism were negatively correlated. The success associated with balance can perhaps best be achieved by having supporters on both sides of the continuum, espousing views associated with one or the other side of the political spectrum, as reflected in the Social Order versus Social Justice perspectives of conservatives versus liberals respectively. This study provides some early, perhaps tentative evidence that the two group moralities may serve as counterweights for the benefit of society.

## General Discussion

Although liberals and conservatives share a common morality in the intrapersonal and interpersonal domains, they diverge when it comes to group-based moral motives. Here conservatives emphasize Social Order, whereas liberals stress Societal Justice. Libertarians are low on both binding group moralities; it appears they believe societal decisions should be based on individual autonomy across the board rather than collective concerns. Conservatives’ Social Order morality is reflected in tight societies, with their emphasis on strict norm adherence and relative intolerance of behavioral deviance. Liberals’ Social Justice morality, in contrast, seems more closely associated with loose societies, with their emphasis on normative openness and their greater association with egalitarianism. Interestingly, societies that balance tightness and looseness appear to be those that experience the greatest success, as assessed by both economic outcomes and subjective well-being in the current research. Based on these findings, it is intriguing to entertain the possibility that societies with strong advocates on both sides of the political spectrum may succeed in part because of the balance afforded by their opposition.

Morality facilitates social living; it helps us live together in interdependent groups. This functional perspective suggests that distinct moral motives are likely to address different societal challenges. A group morality based in Social Order is likely to be particularly effective in response to threats that require coordinated actions, whereas a group morality based in Social Justice is likely to be particularly effective when cooperative efforts are called for to improve group welfare. Not surprisingly, then, in recent work we found that Social Justice is associated with trust of unknown others, which is required for cooperative efforts; in contrast, Social Order is associated with generalized distrust, and thus the need for strong leadership and loyalty (i.e., MFT binding foundations) in the service of conformity and order [49]. There are likely to be costs and benefits associated with either orientation alone. A Social Order orientation may minimize risks associated with free riding and social loafing, whereas a group morality based in Social Justice may minimize the lost opportunities apt to be associated with the transaction costs of Social Order.

Some recent work in political science has argued that political ideologies are in our DNA [23,50]. It seems questionable that such broad ideologies are genetic, but it is certainly possible that motivational differences that we suggest may underlie these ideologies—and are reflected in distinct group moralities—may have some basis in basic temperament and physiology. Work reviewed above on increased reactivity to threat by conservatives [20,28,32], for example, is suggestive of such a perspective. Yet the differences reflected in endorsement of Social Order versus Social Justice are surely apt to be the result of multiple factors involved in socialization and life experience. Thus, in our own research we found that restrictive parenting was positively associated with a grown child’s Social Order orientation [26]. Such parenting focuses on proscriptions (should nots) and tries to control the child’s behavior through strict limits, threats, and punishment. Lenient parenting, the opposite of restrictiveness, was not associated with Social Justice (and nurturant parenting was not associated with either group morality). However, leniency is unlikely to describe the openness that accounts for greater inclusion of others, a feature of Social Justice [2,3]. Greater acceptance of difference—and of others who are different—may underlie Social Justice and suggests the importance of life experience. It is not surprising, for example, that large cities, with their tremendous diversity, are generally politically liberal, and reported above, Social Justice is strongly correlated with liberalism. In 2012, 27 of the 30 most populous cities in the U.S. voted Democratic; even every one of Texas’ major cities—Austin, Houston, Dallas, and San Antonio—all voted Democratic in the midst of a very Republican state [51]. There is certainly self-selection in terms of who chooses to live in an urban area, reflecting a pre-existing openness to broader experience, but it is also likely that the experience of living in a city has a further impact as well. In the end, it is likely that multiple factors, from temperament and personality to parents and other socializing agents (e.g., other family members, teachers, friends) to lived experiences, contribute to an individual’s support for Social Order or Social Justice and political orientation more generally. In recognizing the benefits of balance in group moralities suggested by the current research, we were drawn to results of Presidential elections in the United States. An interesting feature of these contests is just how close they are in every four-year cycle; that is, we have been struck by the marked balance in left-right voting in these national elections. In the past 50 years, averaged across elections since 1964 (i.e., 13 Presidential elections), 51.76% of the percent of the popular vote has gone to the winning Presidential candidate, and when we consider all Presidential elections between 1828 and 2012 (47 elections), the average percent of the popular vote going to the winning candidate changes very slightly to 51.80% [52]. (The popular vote was not recorded prior to 1824, so the first nine Presidential elections are not included. We have also not included the 1824 election, because John Quincy Adams won with 30.92% of the vote. If this election is included, the average percent popular vote from 1824 through 2012 is 51.36%.)

Partisans on one side of the political spectrum no doubt find it hard to believe there are so many people on the other side. Yet election cycle after election cycle, with very few exceptions, some sort of equilibrium seems to be operating in the electorate, with liberals and conservatives, Democrats and Republicans, voting in relatively equal numbers for the candidate of their choice. Rather than cause for consternation or concern, perhaps this pattern should be considered an implicit indicator of societal success.

Those of us with strong political views on one side or the other no doubt regard this view of adaptive balance as unpalatable. The nature of morality, including group morality (i.e., Social Order and Social Justice), is such that we regard our own convictions as absolute, objective and necessarily right [53]. We thus feel certain that a society based wholly on our own morality would surely be best. Yet the reality on the ground is that we maintain our own moral perspective at the same time that others are pushing for opposing positions [54], balancing our own views. It is this system of countervailing views that may be adaptive in the end.

No doubt the current political paralysis in the U.S. would lead to the opposite conclusion—that is, that the opposing group moralities are clearly maladaptive in a single political system. We would suggest, however, that this is attributable not to the group-based moralities per se, but to a two-party system bent on electoral success rather than societal success. Politics requires negotiation and compromise, conciliation and concessions on the path to middle ground. The group moralities are socially evolved orientations that contribute to group survival across varied historical and ecological circumstances. Perhaps we would all benefit from recognizing that our political positions are not only conflicting, but ultimately complementary when viewed from the grander perspective of long-term societal success.

## Appendix: Model of Moral Motives (MMM) Scale

### Self-Restraint

It’s particularly important to me to demonstrate self-control in the face of temptation.

Exercising self-discipline is an important way for me to feel like a decent person.

It’s not always easy to avoid temptations, but for my own good I feel I really have to try my best.

Life is full of unhealthy attractions, so it’s necessary for me to develop a strong sense of self-discipline and control.

It’s important for me to avoid overindulging when it comes to life’s pleasures.

### Industriousness

I consistently put the necessary time and effort into providing for my own well-being and success.

I value hard work and personal commitment when it comes to making decisions in my life.

When things get tough, I apply myself and work even harder to overcome difficulties.

I think it’s important to take responsibility for my failures and setbacks rather than blame other people.

Whether or not I have others to lean on, I think it’s important for me to try to provide for myself.

### Not Harming

A fundamental rule I live by is “do not cause harm.”

I especially dislike people who cheat to get ahead.

We should never steal from other people.

It is always wrong to kill another human being.

There is no excuse for taking advantage of others for one’s own gain.

### Helping/Fairness

When someone does me a favor, I try particularly hard to return the favor.

Having compassion for someone who is suffering is an extremely admirable trait.

Treating others fairly is a clear sign of a good person.

A decent person will go out of his or her way to help others.

Being generous is an important part of who I am.

### Social Order

In a good society, there must be very little deviation from behaviors viewed as appropriate.

It is harmful to society when people choose radically new lifestyles and ways of living.

There are good reasons why traditional ways of living have lasted for so long, even if people don’t fully understand those reasons.

In a decent society, people should strictly attend to the values and practices of the larger community.

The best societies are usually the least permissive societies.

### Social Justice

It is our responsibility, not just a matter of personal preference, to provide for groups worse off in society.

Giving to groups worse off in society does not make those groups too dependent on help.

It is important for those who are better off to help provide resources for the most vulnerable members of society.

Increased economic equality is ultimately beneficial to everyone in society.

In the healthiest societies, those at the top should feel responsible for improving the well-being of those at the bottom.

## Supporting Information

S1 File Study 1 Data. (SAV)

S2 File Study 2 Data. (SAV)

S3 File Study 3 Data. (SAV)
